# Retraction: Food security management in developing countries: Influence of economic factors on their food availability and access

**DOI:** 10.1371/journal.pone.0330117

**Published:** 2025-08-14

**Authors:** The PLOS One Editors

After this article [1] was published, concerns were identified about competing interests and peer review. PLOS consulted a member of the *PLOS One* Editorial Board, who noted additional concerns regarding the study design, methodology, reporting, interpretation of results and support for conclusions:

- The article [1] contains citations that do not support the statements in which they are cited, specifically references 42 and 43 in the Background of the food security approach section.
- The sample size (n = 19 per country) is relatively small for multivariate modelling and no robustness checks or time-series diagnostics are reported in [1].
- Food Production Index (FPI) is used as a dependent variable to measure food security in [1] but FPI does not capture access, a key domain in food security. Access-related indicators (poverty, inflation) are used to explain a production-related outcome in [1].
- Confidence intervals and regression diagnostics are not shown.
- Claims in [1] that poverty determines production may be reverse causality, and [1] does not include models to verify directionality.
- Several data values appear interpolated or imputed without clear explanation.
- The regression models use percentages without adjusting for GDP size or food import value, which introduces scaling bias.
- The article presents generalized and overstated conclusions that are not supported by the study design of two case studies without consideration of potential bidirectional relationships.
- The policy suggestions in [1] are not sufficiently anchored in the data presented.

The first author provided the following clarifications:

- Timeseries diagnostics were not performed due to data constraints.
- FPI was chosen as a proxy for food availability and does not fully capture access-related dimensions, which is a limitation of [1], but the inclusion of economic access variables in the model offers partial insights.
- FPI reflects aggregate volume, not nutritional quality or food affordability and therefore, conclusions based on FPI in [1] relate only to physical availability.
- The regression results reflect associations rather than causality and models to verify directionality were not applied in [1] due to sample limitations.
- Some missing data, particularly in the Kyrgyz inflation series, were linearly interpolated which is a limitation of [1].

Following editorial assessment and consultation with a member of the journal’s Editorial Board, the *PLOS One* Editors concluded that the article does not meet the journal’s criteria for publication.

In light of the above concerns, the *PLOS One* Editors retract this article [1]. We regret that the issues were not identified prior to the article’s publication.

LV and JPCT did not agree with the retraction. NB did not respond to the final editorial decision. BS and RM either did not respond directly or could not be reached.
